# Knockdown of WAVE3 impairs HGF induced migration and invasion of prostate cancer cells

**DOI:** 10.1186/s12935-015-0203-3

**Published:** 2015-05-10

**Authors:** Muhammad Moazzam, Lin Ye, Ping-Hui Sun, Howard Kynaston, Wen G Jiang

**Affiliations:** Metastasis & Angiogenesis Research Group, Department of Surgery, Institute of Cancer and Genetics, Cardiff, UK; Cardiff China Medical Research Collaborative, Cardiff University School of Medicine, Cardiff, CF14 4XN UK

**Keywords:** WAVE3, HGF, Prostate cancer, Invasion and migration

## Abstract

**Background:**

The WASP (Wiskott-Aldrich syndrome protein) and WAVE (WASP Verpolin homologous) family of proteins are structurally related and responsible for regulation of actin polymerization through their interaction with actin related proteins 2&3 (ARP 2/3). WAVE-3 has exhibited an association with disease progression and poorer prognosis of certain malignancies. In the current study, we determined the role of WAVE-3 in hepatocyte growth factor induced cellular changes including cell matrix interaction, invasion and cellular motility, and pathways that may be responsible for the changes in prostate cancer cells.

**Methods:**

We used hammer head ribozymes to knock down the expression of WAVE-3 in PC-3 prostate cancer cell line. In vitro cellular functional assays including growth, invasion, adhesion, motility and invasion, were performed to assess the effects of WAVE-3 knock down. Further experimentation was performed to investigate the role of different pathway through expression and phosphorylation status of various intermediate proteins.

**Results:**

WAVE-3 knockdown reduced invasive potential and motility of prostate cancer cells. Following addition of HGF, control cells showed significantly increased invasion and motility (*p* value <0.5) and marked increase in cellular growth. However, WAVE-3 knockdown cell line failed to show any increase in these trends (*p* value <0.5) except increased growth compared with control cells. Further experiments revealed that HGF-induced activation of Paxillin was weakened by the knockdown of WAVE-3. Our study also indicated that reduced invasiveness following WAVE-3 knockdown, may be related to reduce activity of MMP-2.

**Conclusions:**

Our studies suggest a vital role of WAVE-3 in HGF induced invasion and migration in which Paxillin and MMP-2 are involved. Further study will shed light on its potential as therapeutic target to suppress local invasion and metastasis of prostate cancer cells.

**Electronic supplementary material:**

The online version of this article (doi:10.1186/s12935-015-0203-3) contains supplementary material, which is available to authorized users.

## Background

Prostate cancer is the most commonly diagnosed male malignancy in the US and UK [1, 2]. Morbidity and mortality is mainly related to metastatic disease [3]. Understanding the mechanism and molecules involved in creation of metastatic disease will help in devising the treatments and metastatic prevention strategies.

Invasive and metastatic potential of any cancer is dependent upon expression of different cellular characteristics at various steps of the metastatic cascade. Cellular motility and cytoskeleton changes are integral parts of this process and depend upon actin polymerization. Cells exhibit these changes in response to interaction with the external environment through surface proteins. Integrins is one such class of proteins responsible for regulating the binding of a cell to components of the external environment. Paxillin, through tightly regulated interactions with multiple structural and signaling molecules, serves as a nexus for the control of the Rho family of GTPases which act as essential regulators of the actin polymerization [4, 5]. Paxillin contributes to the regulation of the Rho family of GTPases by indirectly recruiting various GEFs (guaninenucleotide–exchange factors), and GAPs (GTPase activating proteins) which promote the hydrolysis of GTP to GDP [6]. It has been observed that an increase in Paxillin phosphorylation correlates to the metastatic ability of the cell [7]. Phosphorylation of Paxillin in metastatic cells is influenced by numerous growth factors [8].

Hepatocyte growth factor (HGF) utilises one such pathway to mediate cell-matrix adhesion and has been shown to enhance paxillin phosphorylation [9]. HGF derived from prostate stroma, promotes proliferation, differentiation, motility, and invasion of malignant epithelial cells indicating possible involvement in the progression of prostate cancer [10]. The serum levels of HGF and PSA are found to be significantly increased in prostate cancer patients [11]. Higher plasma levels of HGF in men with hormone-refractory prostate cancer are associated with a decreased patient survival [12].

The WASP (Wiskott-Aldrich syndrome protein) and WAVE (WASP Verpolin homologous) family of proteins are structurally related and responsible for regulation of actin polymerization through their interaction with actin related proteins 2&3 (ARP 2/3) [13]. WASP family includes WASP and N-WASP. WAVE family comprise of WAVE-1, 2 and 3. WASP and WAVE family act as downstream regulators of small GTPase like Rho and Rac responsible for signal transduction generated by different regulatory factors. Rac (GTPase) controls the formation of lamellipodia through regulation of WAVE family. WAVE-1 appears to be crucial for dorsal ruffles, while WAVE-2 is necessary for formation of lamellipodia. WAVE-3 was found to be an aetiological factor in the development of low grade neuroblastoma [14]. Prior studies have shown that elimination of WAVE-3 from breast and prostate cancer cells reduces their invasive potential through reduction in motility and reduced expression of enzymes responsible for extra cellular matrix degradation [15-17].

HGF can increase both invasion and haptotactic migration of prostate cancer cells [18]. Previous studies have clearly demonstrated the loss of aggressive phenotype in different cancer cells following elimination of WAVE-3, including prostate cancer cells [16, 19]. This study examined the effect of WAVE-3 on the HGF induced migration and invasion of prostate cancer cells.

## Materials and methods

### Materials

PC-3 cells (European Collection of Cell Cultures, Salisbury, United Kingdom) were maintained in Dulbecco’s modified Eagle’s (DMEM)-F12 medium supplemented with 10 % fetal calf serum and antibiotics. Anti-WAVE3 antibody was obtained from Santa Cruz Biotechnology, Santa Cruz, CA. Mouse monoclonal anti-Paxillin antibody was purchased from BD Bioscience. Other kits and reagents were obtained from Sigma-Aldrich, Poole, UK. The recombinant human HGF/SF was from HGF/SF cDNA transfected Chinese hamster ovary cell and was a kind gift from Dr. T. Nakamura, Osaka, Japan.

### Construction of anti-WAVE3 ribozyme transgenes, and transfection

Anti-WAVE-3 ribozyme transgenes were synthesised and then cloned into pEF6/V5-His-TOPO-TA plasmid vector (Invitrogen, Paisley, UK). The anti-WAVE3 transgenes were then used to transfect PC-3 cells following a previously described procedure [19].

### RNA isolation and PCR

RNA was isolated from selected cells using total RNA isolation reagent (ABgene, Surrey, UK). Following quantification, this RNA was used to construct complimentary DNA using DuraScript reverse transcriptase-polymerase chain reaction (RT-PCR) kit (Invitrogen, Paisley, UK). This DNA was used for routine PCR and Q-PCR to confirm the knock down of WAVE-3 and expression of different genes. Details of primers are provided in Table 1.

**Table 1:** PCR primer sequences used in the study

Gene	Sense (5′-′3)	Antisense (5′-′3)
WAVE-3	TACTCTTGCCGCTATCATACG	TGCCATCATATTCCACTCCTG
β-actin	ATGATATCGCCGCGCTCG	CGCTCGGTGAGGATCTTCA
MMP-1	GGATGCTCATTTTGATGAAG	TAGAATGGGAGAGTCCAAGA
MMP-2	TTTGATGACGATGAGCTATG	TGCAGCTCTCATATTTGTTG
MMP-7	GCTATGCGACTCACCGTGCTGTG	AGCGTGTTTCCTGGCCCATCAAATG
MMP-9	AACTACGACCGGGACAAG	ATTCACGTCGTCCTTATGC
MMP-11	GTGCCCTCTGAGATCGAC	CAGGGTCAAACTTCCAGTAG

### Western blot

Equal amount of proteins were separated from cell lysate using DC Protein Assay kit (Bio-Rad Laboratories, Hemmel Hempstead, UK) and an ELx800 spectrophotometer (Bio-Tek, Wolf Laboratories, York, UK). These proteins were separated using Sodium dodecyl sulfate-polyacrylamide gel electrophoresis (SDS-PAGE) and blotted on to nitrocellulose membrane. Membrane was treated with milk to block non-specific proteins prior to probing with anti-WAVE-3 primary antibodies, followed by peroxidase conjugated secondary antibodies. Protein bands were visualized and analysed using Supersignal West Dura system and documented using a gel documentation system (UVITech, Cambridge, United Kingdom).

### Immunoprecipitation for detection of paxillin phosphorylation

The cells were cultured in serum free medium for one hour, followed by treatment with HGF containing serum free medium for 30 min. Following this, the cells were harvested and lysed. Tyrosine phosphorylated proteins were immunoprecipitated using PY20 antibody (1:500 dilution) and protein A/G agarose beads (Santa Cruz Biotechnology, Calne, UK). Immunoprecipitates were then separated by SDS-PAGE (10 % gel), electroblotted on to nitrocellulose membrane and probe with specific antibodies.

### Zymography

Gelatinase zymography was performed in 8 % SDS Polyacrylamide Gel in the presence of 0.1 % gelatin under non-reducing conditions [20]. Upon renaturation of the enzyme, the gelatinases digest the gelatin in the gel and give clear bands against an intensely stained background. Gels were subsequently scanned on digital scanner and digital images were saved for subsequent analysis.

### Cell growth assay

Cell growth was assessed using previously described method [21]. In this method, 3000 cells are added each well in 96 well plates and growth was assessed after 72 and 120 h, in the presence or absence of HGF (40 ng/ml) containing medium. Crystal violet was used to stain the cells, which was extracted and subsequently used to quantify through absorbance of light (wave length 540 nm) using an ELx800 spectrophotometer. Amounts of absorbance represented the number of cell and change was calculated by percentage increase as compared to baseline reading.

### Cell matrix adhesion assay

Cells were added to a Matrigel precoated (5 μg/well) 96-well plate (30,000/well). Cells were incubated for 40 min with or without HGF/SF (40 ng/ml). Non-adherent cells were washed with BSS buffer. Adherent cell were stained with crystal violet (200 μL/well) and then documented.

### Invasion assay

Transwell inserts with pore size of 8 μm were precoated with Matrigel (50 μg/well, BD Biosciences, Oxford UK) and used for invasion assay as previously described [22]. Cells were added along with medium (20,000 cells/insert). Experiment was run in duplicate for each cell line with or without HGF/SF (40 ng/ml). After 72 h of incubation, the number of cells that have invaded through the basement membrane were counted following staining with crystal violet solution as described above in adhesion assay.

### Migration assay

PC-3 cells were seeded into a 24-well plate (250,000 cells/well) and allowed to form a monolyer by an overnight culture. The cell monolayer was scraped using a sharpened 200 μl plastic tip. Closure of the wounds was photographed. Migration of the cells was measured using ImageJ software (National Institutes of Health, USA).

### Cell motility assay using cytodex-2 beads

Cell motility was assessed by dissociation from cytodex-2 μ carrier beads following with or without treatment by HGF/SF (40 ng/ml) and was adapted from previous published methods [23]. Cells at a density of 1 × 10^6^ cells/ml were cultured in 10 ml of DMEM medium containing 250 μg/ml of beads for 24 h at 37 °C. The cell/bead complex was seeded into a 24 well plate in triplicate, and cultured with (HGF 40 ng/ml) or without HGF for 4 h at 37 °C. Migrated cells were counted following the fixation and staining with crystal violet.

### Immunofluorescent staining

Following the treatment to the cells, immunofluorescent staining was performed using primary antibody and secondary antibody (labelled with FITC). Cells were viewed under fluorescent microscope (Olympus) and photos were taken.

### Data analysis

Mann Whitney test was initially used to check the normal distribution of data, while paired sample *t*-test was used to compare the different groups for finding significant difference. These results were also analysed using SPSS version 12.0.1 (Chicago, IL). Differences were considered statistically significant at p <0.05.

## Results

### Knockdown of WAVE-3 in PC-3 cells and the effect on cell growth

**Fig. 1:**
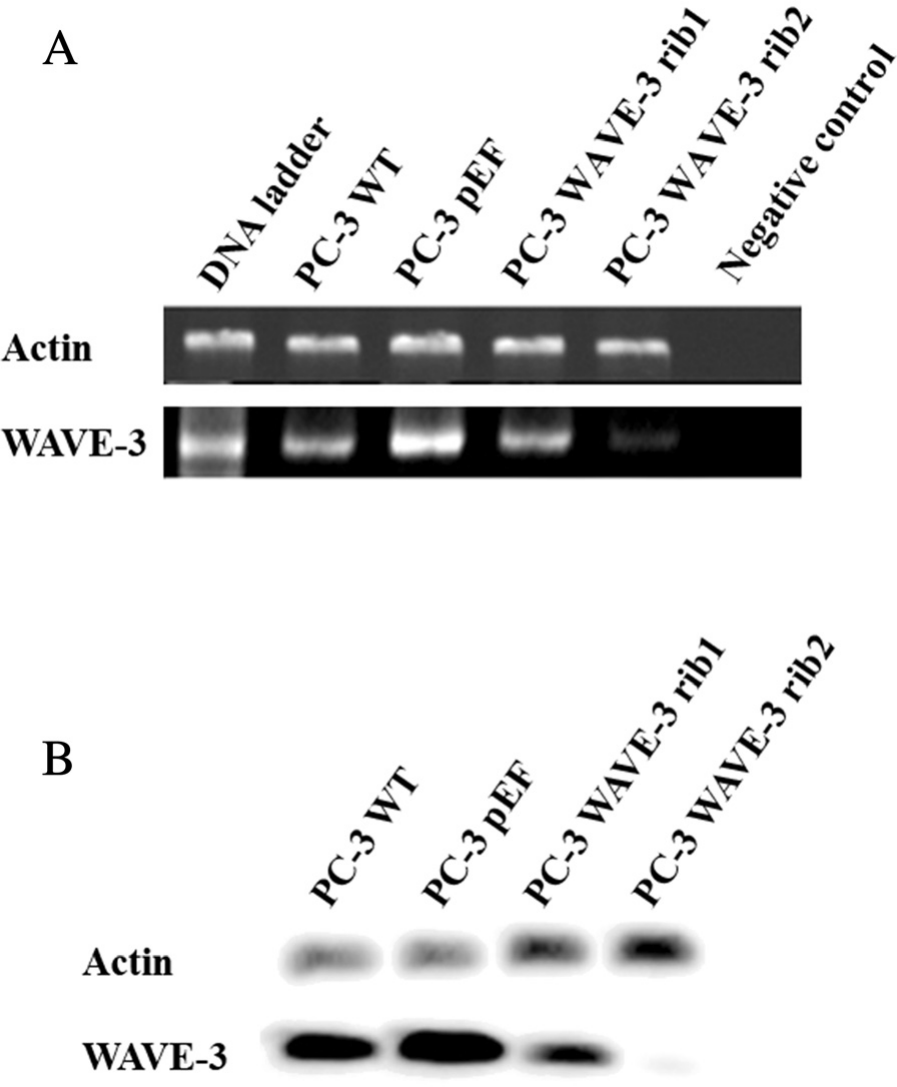
Knockdown of WAVE-3 in PC-3 cells using anti-WAVE3 ribozyme transgenes. **a**. RT-PCR shows the WAVE-3 transcript. **b**. Western blot reveals the WAVE-3 protein. Total RNA and protein were extracted from the cells within the first two weeks after the selection. The cells were maintained medium with 0.5 μg/ml blasticidin. Three independent experiments were carried out for a confirmation of the knockdown using the anti-WAVE-3 ribozyme transgenes

Cells transfected with the anti-WAVE-3 ribozyme-2 transgene (PC-3 ΔWAVE-3 Rib2) exhibited a markedly reduced level of WAVE-3 expression compared controls and cells transfected with the anti-WAVE-3 ribozyme-1 transgene (PC-3 ΔWAVE-3Rib1) (Fig. 1). There was no change observed in expression of WAVE 1 and 2 following WAVE-3 knock down (data not shown).

**Fig. 2:**
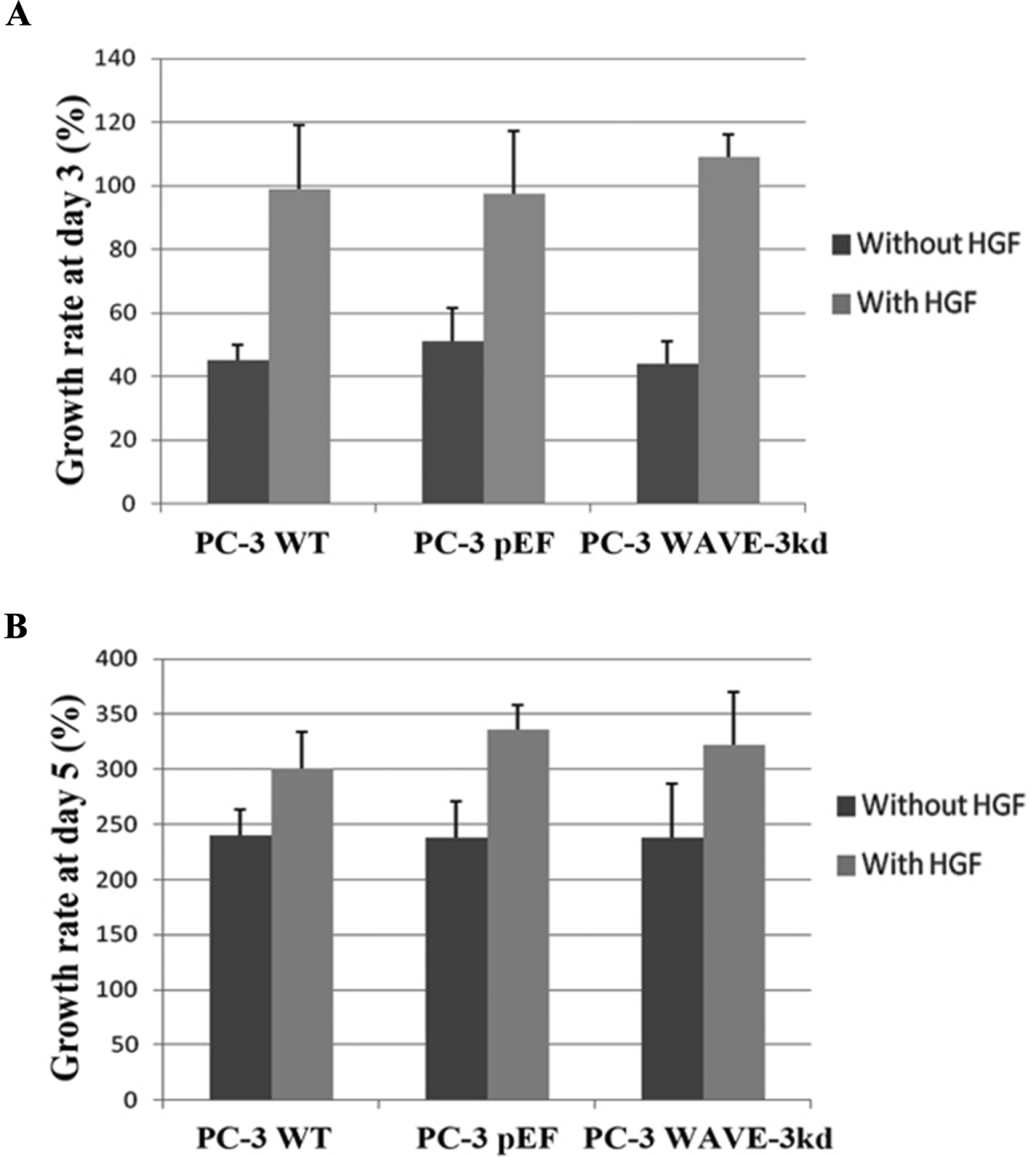
WAVE-3 knockdown and HGF regulated in vitro growth of PC-3 cells. **a**. The cell growth was measured after 3 days’ culture. **b**. The growth over 5-day period. Growth rate was calculated with a formula: (Absorbance Day3 –Absorbance Day1)/Absorbance Day1 *100 (%). Shown are representative results of three independent experiments performed. Error bars stand for standard deviations

WAVE-3 knock down did not demonstrate any effect on growth of PC-3 cells at 72 h and at 120 h (Fig. 2). There was marked increased growth with addition of HGF (40 ng/ml), particularly at 72 h. The growth response between the different cells was not statistically significant.

### Effect of WAVE-3 knock down on cellular matrix adhesion and invasion

Elimination of WAVE-3 did not significantly affect the cell adhesion with matrix. With the addition of HGF (40 ng/ml), there was increase in adhesive potential of cells but there was no statistical difference between the various cell lines (Data not shown).

**Fig. 3:**
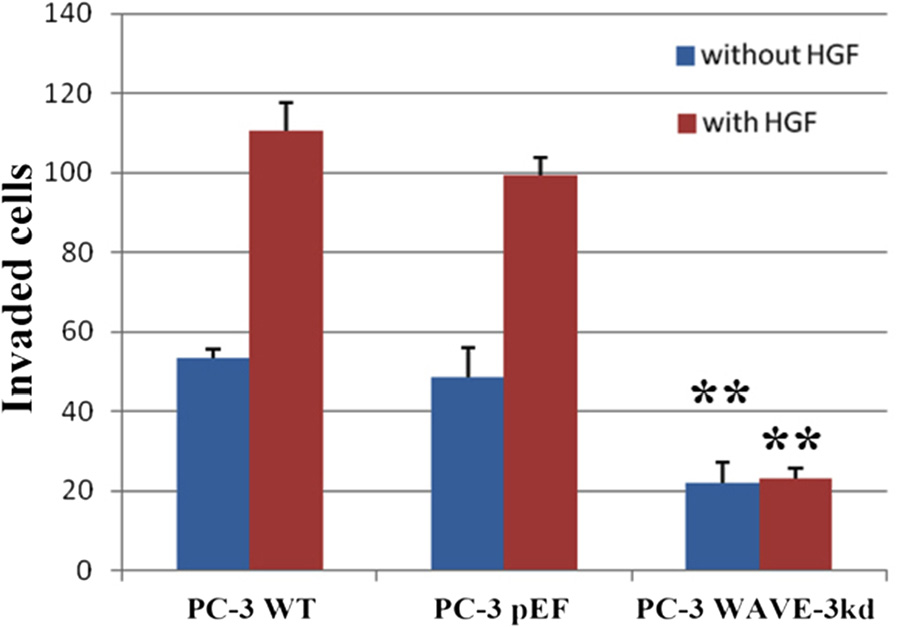
Effect of WAVE-3 on the HGF regulated cell invasion. WAVE-3 knockdown impairs the HGF induced invasion of PC-3 cells. The mean+/−SD number of cells invaded in PC-3WAVE-3KD was 22.10+/−5.13 as compared to PC-3WT 53.23+/−2.5 and PC-3pEF 48.64+/−7.33 respectively (*p* value <0.05). Shown are representative results of three independent experiments performed. Error bars stand for standard deviations

WAVE-3 knockdown in PC-3 cells significantly decreased the invasion of cells through the matrigel basement membrane (Fig. 3). There was significant decrease in the invasiveness of PC3WAVE-3KD cells as compared to PC-3WT and PC-3pEF cells. The addition of HGF 40 μg/ml to the medium resulted in stimulation to invasive potential of different PC-3 cells to variable degree except the PC-3WAVE-3KD cells. In addition it was also observed that invaded cells in PC-3WT and PC-3pEF types were significantly higher than PC-3WAVE-3KD, even after the addition of HGF induced stimulation.

### Effect of WAVE-3 knock down on cellular motility

**Fig. 4:**
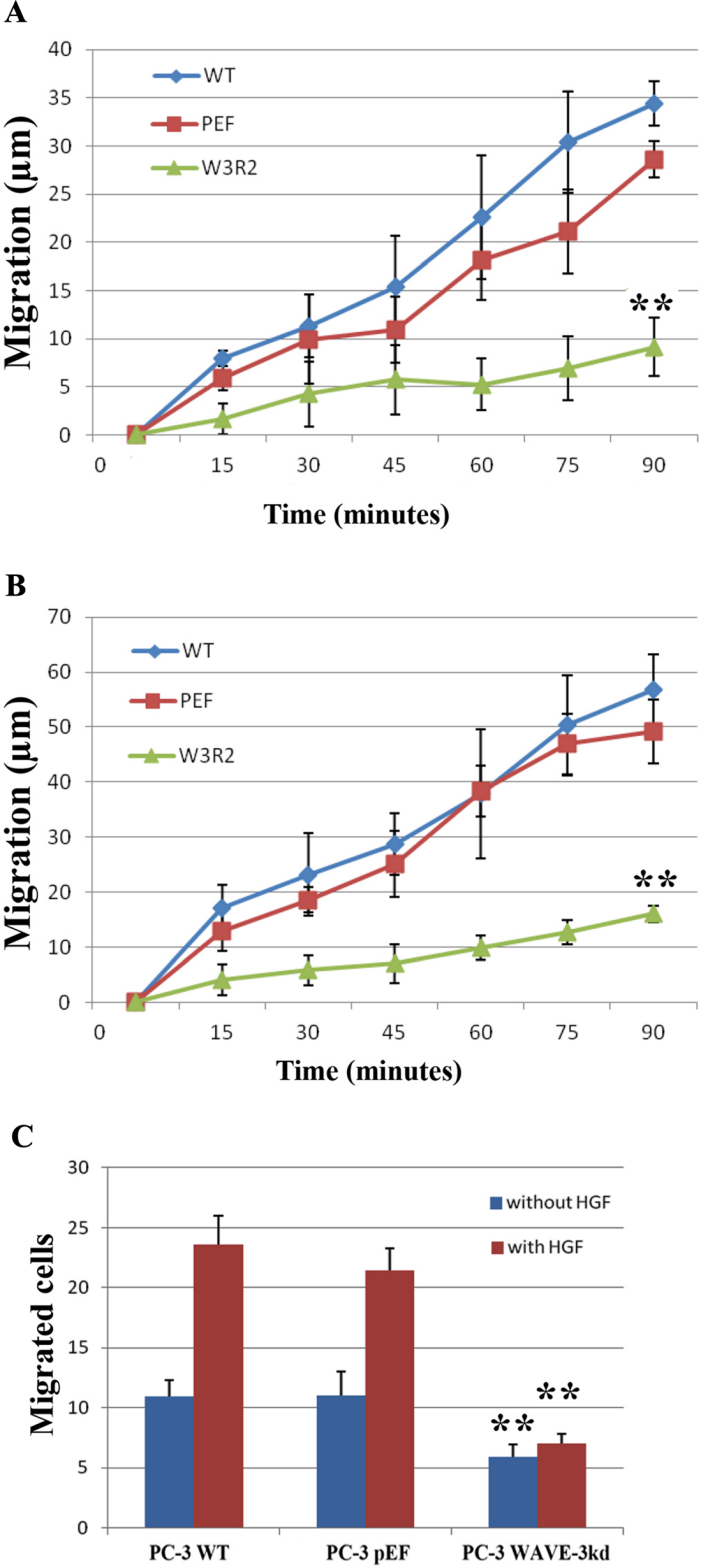
WAVE-3 and HGF promoted cell migration of prostate cancer cells. **a**. Migration of PC-3 cells was determined using wounding assay. **b**. Effect on HGF promoted migration of prostate cancer cells by WAVE-3 knockdown were assessed using wounding assay. **c**. WAVE-3 knockdown and HGF regulated cell migration was also assessed using Cytodex-beads motility assay. Shown are representative results of three independent experiments performed. Error bars stand for standard deviations

We also determined the influence on cell migration using wounding assay (Fig. 4a and b). Knockdown of WAVE-3 resulted in a decrease of migration in PC-3 cells and led to a reduced response to HGF stimuli. In line with the findings of wounding assay, cytodex-2 bead motility assay showed that elimination of WAVE-3 resulted in reduction of motile cells (Fig. 4c). HGF promoted the motility of the control cells but failed to trigger the similar response in the WAVE-3 knockdown cells.

### Effect of WAVE-3 elimination on paxillin and its HGF induced phosphorylation

To investigate the underlying molecular mechanism, we analyzed the expression of Paxillin, FAK and ERM proteins along with their phosphorylation status following elimination of WAVE-3 and response to addition of HGF. No obvious difference was observed for both expression and distribution of FAK and ERM in the WAVE3 knockdown cells (Additional file 1: Figure S1, Additional file 2: Figure S2, Additional file 3: Figure S3, Additional file 4: Figure S4).

**Fig. 5:**
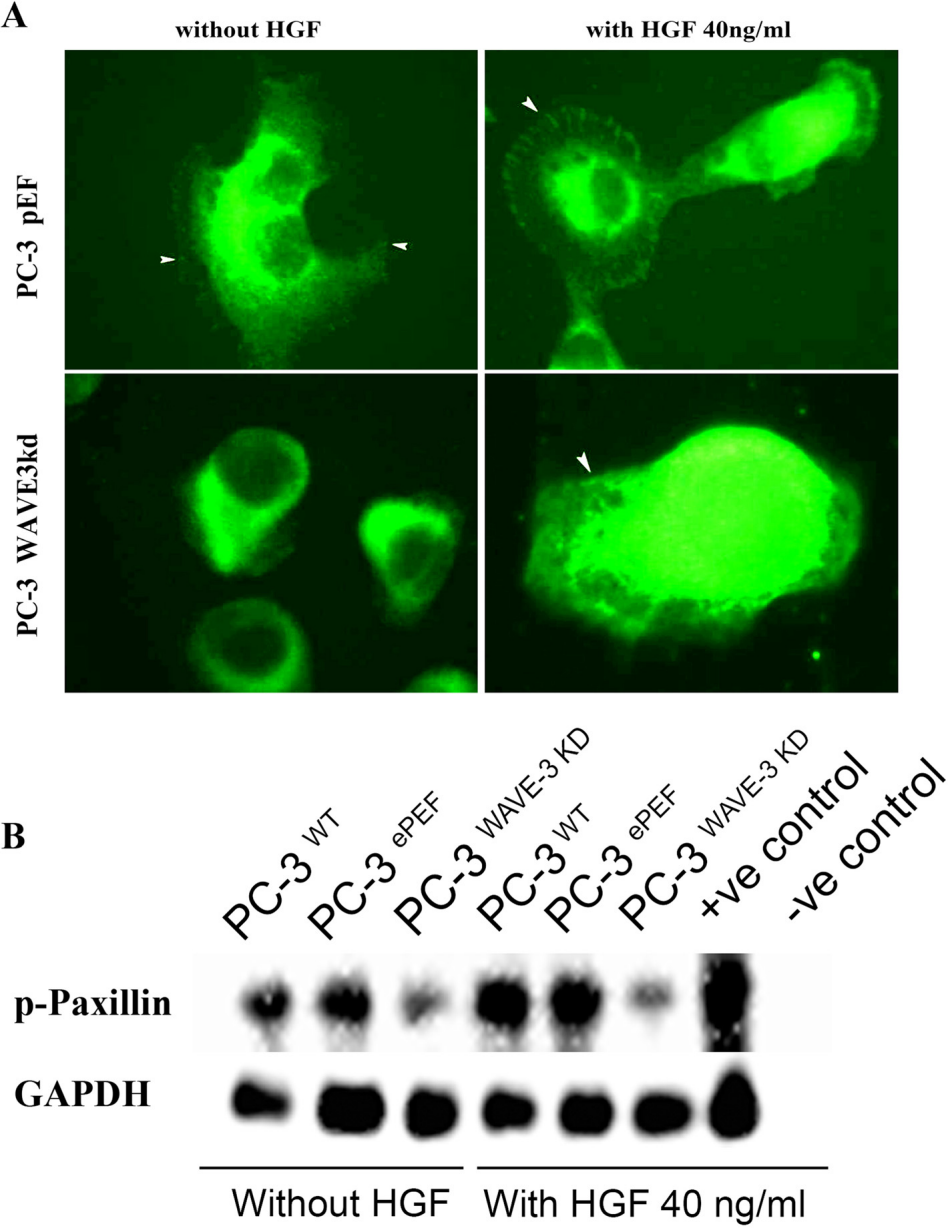
Paxillin in the WAVE-3 knockdown PC-3 cells. **a**. Distribution of Paxillin in the WAVE-3 knockdown cells was examined using immunofluorescent staining by anti-Paxillin antibody (BD Biosciences, 610051). Arrows point to enhanced staining of Paxillin at the focal adhesions which was not observed in the WAVE-3 knockdown cells. **b**. Activation of Paxillin in the PC-3 was determined using immunoprecitation and Western blot. Tyrosine phosphorylated proteins were immunoprecipitated using PY20 (p-Tyr antibody, Santa Cruz Biotechnology, SC-508). GAPDH was used to probe cell lysates which were the same input samples for the immunoprecipitation as loading control

Expression and phosphorylation of these proteins was detected using immunoprecipitation and subsequent western blotting. In our experiment, good expression of paxillin was observed in all cells. There were no changes in the expression of Paxillin after WAVE-3 knock down. However the levels of phosphorylated Paxillin were observed to be reduced. When cells were treated with HGF (40 ng/ml for 30 min), there were increased levels of phosphorylated Paxillin (Fig. 5b) in PC-3^WT^ and PC-3^pEF^ cells, but PC-3^WAVE-3KD^ cells failed to show such response. Immunofluorescent staining of all three cells showed prominent fluorescence with Paxillin (Fig. 5a). With addition of HGF there was enhanced staining of Paxillin at the focal adhesions of PC-3WT and PC-3pEF cells. However PC-3WAVE-3KD cell line failed to exhibit such response following addition of HGF.

### Knockdown of WAVE-3 influences MMPs activity in PC-3 cells

**Fig. 6:**
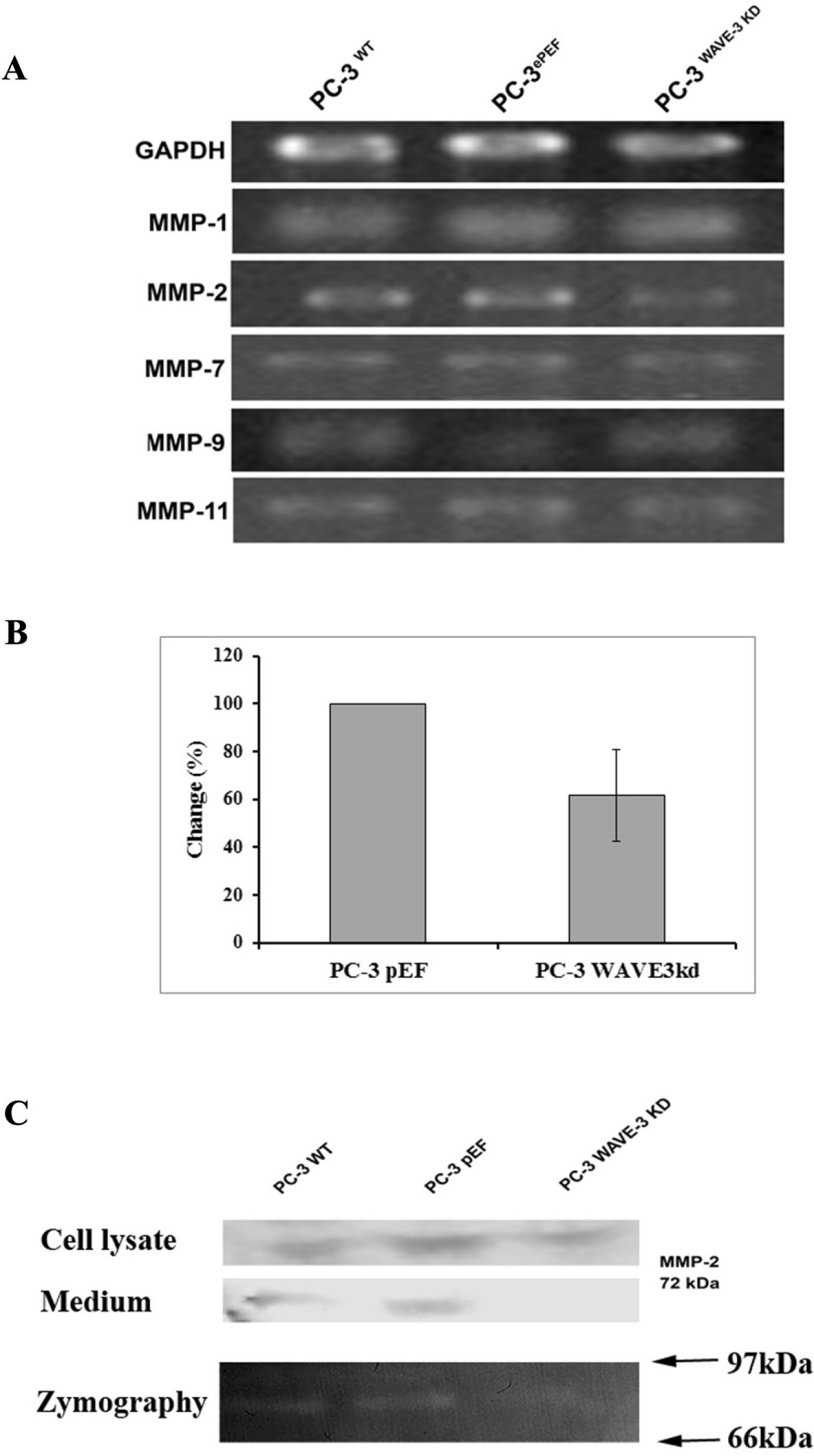
Knockdown of WAVE-3 reduced MMP-2 activity in the PC-3 cells. **a**. Expression of MMPs was examined using RT-PCR. **b**. Semi-quantification was performed to determine the change of MMP2 transcripts in WAVE-3 knockdown cells compared with PC-3pEF control cells. Integrated density of the MMP2 bands was normalised against the corresponding GAPDH bands, shown are changes of MMP2 in percentage against its quantity in the control cells. **c**. MMP-2 in cell lysate and culture medium was determined using Western blot (top two panels), while the activity of the secreted MMP-2 (culture medium) was assessed using zymography (bottom panel)

We screened our three cells for expression of MMP-1, MMP-2, MMP-7, MMP-9, and MMP-11. There was no difference in expression of different MMPs except MMP-2 (Fig. 6a and b). MMP-2 expressed at a relatively low level in PC-3^WAVE-3KD^ cells. Considering the role of gelatinases in prostate cancer invasion, we also analysed the expression of MMP-2 and MMP-9 in the medium using western blotting which showed reduced expression of MMP-2 in the medium collected from PC-3^WAVE-3KD^ cells. This is consistent with the finding from gelatin zymography in which MMP-2 activity was significantly reduced in the medium extracted from PC-3WAVE-3KD cells compared with the control cells (Fig. 6c).

## Discussion

Role of Hepatocyte growth factor/scatter factor (HGF/SF) is well established in the metastatic process through its effects on proliferation, dissociation, migration and invasion. Higher HGF levels have been demonstrated in hormone refractory prostate cancer and are associated with poor prognosis. Strong expression of WAVE-3 has been documented in prostate cancer specimens and invasive cancer cells from earlier studies in our department [19]. In this study we focused on role of WAVE-3 in mediation of these changes with emphasis on prostate cancer cells. Our present study provides evidence that WAVE-3 has an important role in HGF induced cellular motility and possible metastasis without significant effect on cellular growth. Following genetic silencing of WAVE-3, analysis of functional studies revealed a statistically significant reduction in HGF induced cellular motility as compared to controls. Similar effects were observed in studies involving invasion through the matrigel basement membrane.

Cell-matrix adhesion is one of the key steps in metastasis and is necessary for invasion through matrix in order to progress the metastatic process. These adhesion sites provide important ports for communication of cells with extracellular matrix and integrins play a pivotal role in this process. Paxillin is an important molecule in integrin-mediated adhesion signals and is localized in dorsal ruffles, at the leading edge of cellular movement [24]. An increased degree of Paxillin phosphorylation has been linked to enhanced metastatic potential [25, 26] which indicates its important role in metastasis. Prior studies have documented enhancement of this phosphorylation and localization to the focal adhesion complexes, following addition of HGF in prostate cancer cells [9]. In our study we found no changes in the expression of Paxillin protein following genetic silencing of WAVE-3. Our results clearly indicate that levels of HGF induced phosphorylation were significantly reduced in prostate cancer cells following elimination of WAVE-3 along with reduced motility and invasion. These results suggest a positive feedback mechanism of WAVE-3 on Paxillin phosphorylation. This may serve one of the important mechanisms involved in reduced cellular response to HGF. Reduction in activated phosphorylated Paxillin can result in reduced recruitment of various GEFs, and GAPs/ GTPase activating proteins which promote the hydrolysis of GTP to GDP [6]. These GEFs and GAPs are directly involved in activation of small GTPases including Rac and CDC42. Paxillin is phosphorylated on multiple tyrosine (PY), serine (PS) and threonine (PT) residues in response to cell adhesion, and/or exposure to a various soluble growth factors and cytokines [27]. Tyrosine phosphorylation generates docking sites for SH2 domain containing proteins such as Crk (binds PY31, 118) to facilitate downstream signaling. In contrast, phosphorylation of serine and threonine residues is more likely to influence paxillin conformation and thereby affect its ability to interact with specific binding partners.

Numerous studies have shown that the inhibition of MMPs activity or expression can be a potential target for the prevention of metastasis [28, 29]. MMP-2 and MMP-9 belong to a gelatinase group of MMPs and their role has been extensively studied in prostate cancer [30]. Expression of most MMPs is normally low in tissues, and only induced when remodelling of the extracellular matrix is required. MMP expression is primarily regulated at the transcriptional level, although stabilization of MMP transcripts in response to growth factors, as well as the influence of cytokines, also plays a role in the regulation of MMP activity [31, 32]. The possible involvement of the WAVE proteins in the regulation of MMPs was initially reported by Suetsugu et al., who showed that down regulation of WAVE1, but not WAVE2, affected MMP-2 activity [33]. We further investigated the role of MMPs in reduced invasion of prostate cancer cells following WAVE-3 knock down. Evaluation of expression pattern of different MMPs revealed relatively low expression of MMP-2 on RT-PCR after silencing of WAVE-3. Further evaluation with western blotting and zymography studies showed reduced availability of MMP-2 in the medium. This suggests an important contributory role of MMP-2 in reducing cellular invasion and movement, after WAVE-3 elimination. It is well documented that integrins generate adhesion and induce MMP-mediated ECM degradation [34]. Integrin-mediated adhesion simultaneously activates Rac [35] and a paxillin-associated kinase, FAK [36]. Both Rac and FAK enhance secretion and activation of MMP-2 [37-39]. Actin cytoskeleton itself is involved in regulation of MMPs. Treatment of fibroblasts with cytochalasins increased expression, secretion, and activation of MMP-2 [40, 41]. Our prior studies regarding the role of Paxillin showed markedly reduced levels of phosphorylated Paxillin which may indicate towards crosstalk between these molecules and WAVEs and suggest the differential effects on the MMP-2 and hence on invasion as well. Results of our current study clearly suggest the active role of MMP-2 in reduction of cell motility and invasion following WAVE-3 elimination. However, it needs further investigations to study the role of pathways like ERK1/2 and MAPK/p53, as possible underlying mechanism responsible for these changes.

## Conclusions

In summary, it is safe to conclude that WAVE-3 plays an important role during cellular motility and invasion in prostate cancer cells. It is also actively involved in the mediation of HGF induced changes which possibly promote progression and invasion. Our results strongly point towards the active role of phosphorylated Paxillin and MMP-2 as possible underlying mechanism responsible for these changes. This study provides a part of larger puzzle underpinning the process of metastasis which needs to be further investigated for involvement of other pathways.

## Additional files

Additional file 1: Figure S1. Immunofluorescent staining (IF) of Ezrin in PC-3 cells in exposure to HGF (40 ng/ml) using an anti-Ezrin antibody (Santa Cruz, SC-6409). A TRITC tagged secondary antibody was used for the IF. Arrows point to staining of Ezrin.

Additional file 2: Figure S2. IF of FAK in PC-3 cells in exposure to HGF (40 ng/ml) using an anti-FAK antibody (BD Biosciences, 610087). A FITC tagged secondary antibody was used for the IF. Arrows point to staining of FAK.

Additional file 3: Figure S3. Radixin in PC-3 cells in exposure to HGF (40 ng/ml) was stained using an anti-Ezrin antibody (Santa Cruz, SC-6408) and a FITC tagged secondary antibody. Arrows point to staining of Radixin.

Additional file 4: Figure S4. IF was performed for Moesin in PC-3 cells in exposure to HGF (40 ng/ml) using an anti-Ezrin antibody (Santa Cruz, SC-13122) and a FITC tagged secondary antibody. Arrows point to staining of Moesin.

## Abbreviations

HGF: Hepatocyte growth factor
GEFs: Guaninenucleotide-exchange factors
GAPs: GTPase activating proteins
WASP: Wiskott-Aldrich syndrome protein
WAVE: WASP Verpolin homologous
ERK1/2: Extracellular signal-regulated kinases 1 and 2
MAPK: Mitogen-activated protein kinase
DMEM: Dulbecco’s modified Eagle medium
RT-PCR: Reverse transcription – polymerase chain reaction

## References

[1] Siegel R, Naishadham D, Jemal A (2013). Cancer statistics, 2013. CA Cancer J Clin.

[2] UK CR. Cancer statistics. http://www.cancerresearchuk.org/cancer-info/cancerstats/.

[3] Norgaard M, Jensen AO, Jacobsen JB, Cetin K, Fryzek JP, Sorensen HT (2010). Skeletal related events, bone metastasis and survival of prostate cancer: a population based cohort study in Denmark (1999 to 2007). J Urol.

[4] Brown MC, Turner CE (2004). Paxillin: adapting to change. Physiol Rev.

[5] Price LSLJ, Schwartz MA, Bokoch GM (1998). Activation of Rac and Cdc42 by integrins mediates cell spreading. Mol Biol Cell.

[6] Hoffman GR, Cerione RA (2002). Signaling to the Rho GTPases: networking with the DH domain. FEBS Lett.

[7] Rodina A, Schramm K, Musatkina E, Kreuser ED, Tavitian A, Tatosyan A (1999). Phosphorylation of p125FAK and paxillin focal adhesion proteins in src-transformed cells with different metastatic capacity. FEBS Lett.

[8] Sattler M, Pisick E, Morrison PT, Salgia R (2000). Role of the cytoskeletal protein paxillin in oncogenesis. Crit Rev Oncog.

[9] Parr C, Davies G, Nakamura T, Matsumoto K, Mason MD, Jiang WG (2001). The HGF/SF-induced phosphorylation of paxillin, matrix adhesion, and invasion of prostate cancer cells were suppressed by NK4, an HGF/SF variant. Biochem Biophys Res Commun.

[10] Gmyrek GAWM, Webb CP, Yu HM, You X, Vaughan ED, Vonde Wade GF (2001). Normal and malignant prostate epithelial cells differ in their response to hepatocyte growth factor/scatter factor. Am J Pathol.

[11] Maha Hashem TE (2005). Hepatocyte Growth Factor as a Tumor Marker in the Serum of Patients with Prostate Cancer. J Egypt Natl Canc Inst.

[12] Humphrey PAHS, Picus J, Sanford B, Vogelzang NJ, Small EJ, Kantoff PW (2006). Prognostic significance of plasma scatter factor/hepatocyte growth factor levels in patients with metastatic hormone–refractory prostate cancer: results from cancer and leukemia group B 150005/9480. Clin Genitourin Cancer.

[13] Kang H, Wang J, Longley SJ, Tang JX, Shaw SK (2010). Relative actin nucleation promotion efficiency by WASP and WAVE proteins in endothelial cells. Biochem Biophys Res Commun.

[14] Sossey-Alaoui K, Su G, Malaj E, Roe B, Cowell JK (2002). WAVE3, an actin-polymerization gene, is truncated and inactivated as a result of a constitutional t(1;13) (q21;q12) chromosome translocation in a patient with ganglioneuroblastoma. Oncogene.

[15] Sossey-Alaoui K, Ranalli TA, Li X, Bakin AV, Cowell JK (2005). WAVE3 promotes cell motility and invasion through the regulation of MMP-1, MMP-3, and MMP-9 expression. Exp Cell Res.

[16] Sossey-Alaoui K, Safina A, Li X, Vaughan MM, Hicks DG, Bakin AV (2007). Down-regulation of WAVE3, a metastasis promoter gene, inhibits invasion and metastasis of breast cancer cells. Am J Pathol.

[17] Fernando HS SA, Kynaston HG, Jiang WG. WAVE3 is associated with invasiveness in prostate cancer cells. Urol Oncol. 2009, Epub ahead of print. 10.1016/j.urolonc.2008.12.022 19395286

[18] Fujiuchi YNO, Murakami K, Fuse H, Saiki I (2003). Effect of hepatocyte growth factor on invasion of prostate cancer cell lines. Oncol Rep.

[19] Fernando HS, Sanders AJ, Kynaston HG, Jiang WG (2010). WAVE3 is associated with invasiveness in prostate cancer cells. Urol Oncol.

[20] Ye L, Sun PH, Martin TA, Sanders AJ, Mason MD, Jiang WG (2013). Psoriasin (S100A7) is a positive regulator of survival and invasion of prostate cancer cells. Urol Oncol.

[21] Jiang WG, Davies G, Martin TA, Parr C, Watkins G, Mason MD (2005). Targeting matrilysin and its impact on tumor growth in vivo: the potential implications in breast cancer therapy. Clin Cancer Res.

[22] Jiang WG, Hiscox S, Hallett MB, Scott C, Horrobin DF, Puntis MC (1995). Inhibition of hepatocyte growth factor-induced motility and in vitro invasion of human colon cancer cells by gamma-linolenic acid. Br J Cancer.

[23] Jiang WG, Grimshaw D, Lane J, Martin TA, Abounader R, Laterra J (2001). A hammerhead ribozyme suppresses expression of hepatocyte growth factor/scatter factor receptor c-MET and reduces migration and invasiveness of breast cancer cells. Clin Cancer Res.

[24] Pixley FJLP, Condeelis JS, Stanley ER (2001). Protein tyrosine phosphatase phi regulates paxillin tyrosine phosphorylation and mediates colony-stimulating factor 1-induced morphological changes in macrophages. Mol Cell Biol.

[25] Azuma KTM, Uekita T, Inoue S, Yokota J, Ouchi Y, Sakai R (2005). Tyrosine phosphorylation of paxillin affects the metastatic potential of human osteosarcoma. Oncogene.

[26] Anna Rodina KS, Musatkina E, Kreuser E-D, Tavitian A, Tatosyan A (1999). Phosphorylation of p125FAK and paxillin focal adhesion proteins in src-transformed cells with different metastatic capacity. FEBS Lett.

[27] Burridge KTC, Romer LH (1992). Tyrosine phosphorylation of paxillin and pp125FAK accompanies cell adhesion to extracellular matrix: a role in cytoskeletal assembly. J Cell Biol.

[28] Chakraborti SMM, Das S, Mandal A, Chakraborti T (2003). Regulation of matrix metalloproteinases: an overview. Mol Cell Biochem.

[29] Coussens LMWZ (1996). Matrix metalloproteinases and the development of cancer. Chem Biol.

[30] Zhang LSJ, Feng J, Klocker H, Lee C, Zhang J (2004). Type IV collagenase (matrix metalloproteinase-2 and −9) in prostate cancer. Prostate Cancer Prostatic Dis.

[31] Reunanen NLS, Ahonen M, Foschi M, Han J, Kähäri VM (2002). Activation of p38 alpha MAPK enhances collagenase-1 (matrix metalloproteinase (MMP)-1) and stromelysin-1 (MMP-3) expression by mRNA stabilization. J Biol Chem.

[32] Sasaki MKM, Ito T, Watanabe A, Izumiyama N, Sano M, Kagaya M (2000). Differential regulation of metalloproteinase production, proliferation and chemotaxis of human lung fibroblasts by PDGF, interleukin-1beta and TNF-alpha. Mediators Inflamm.

[33] Suetsugu SYD, Kurisu S, Takenawa T (2003). Differential roles of WAVE1 and WAVE2 in dorsal and peripheral ruffle formation for fibroblast cell migration. Dev Cell.

[34] Friedl PWK (2003). Tumour-cell invasion and migration: diversity and escape mechanisms. Nat Rev Cancer.

[35] Bishop ALHA (2000). Rho GTPases and their effector proteins. Biochem J.

[36] Schlaepfer DDHC, Sieg DJ (1999). Signaling through focal adhesion kinase. Prog Biophys Mol Biol.

[37] Hauck CRHD, Puente XS, Cheresh DA, Schlaepfer DD (2002). FRNK blocks v-Src-stimulated invasion and experimental metastases without effects on cell motility or growth. EMBO J.

[38] Sein TTTA, Hiraiwa Y, Amin AR, Sohara Y, Liu Y, Matsuda S (2000). A role for FAK in the Concanavalin A-dependent secretion of matrix metalloproteinase-2 and −9. Oncogene.

[39] Zhuge Y, Xu J (2001). Rac1 mediates type I collagen-dependent MMP-2 activation. role in cell invasion across collagen barrier. J Biol Chem.

[40] Harris ED, Reynolds JJ, Werb Z (1975). Cytochalasin B increases collagenase production by cells in vitro. Nature.

[41] Tomasek JJHN, Updike DL, Ahern-Moore JS, Vu TK, Liu RW, Howard EW (1997). Gelatinase A activation is regulated by the organization of the polymerized actin cytoskeleton. J Biol Chem.

