# Supplementary figures and images for: Knockdown of WAVE3 impairs HGF induced migration and invasion of prostate cancer cells
Source: Cancer Cell Int. 2015 May 10;15:51. doi: 10.1186/s12935-015-0203-3 (PMC4458333; doi:10.1186/s12935-015-0203-3)

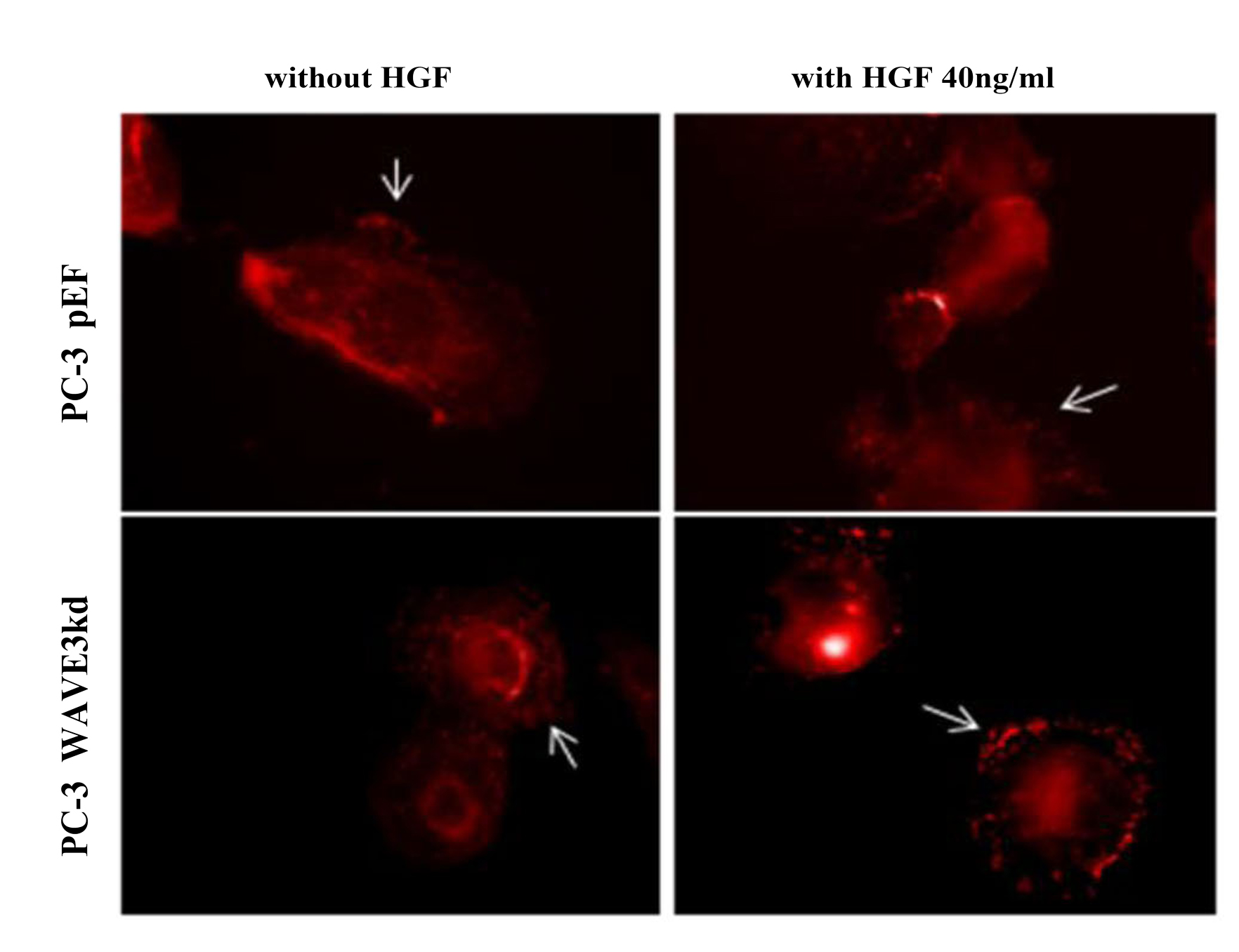

Supplement: Supplementary file 1 — Immunofluorescent staining (IF) of Ezrin in PC-3 cells in exposure to HGF (40 ng/ml) using an anti-Ezrin antibody (Santa Cruz, SC-6409). A TRITC tagged secondary antibody was used for the IF. Arrows point to staining of Ezrin. [file 12935_2015_203_MOESM1_ESM.jpeg]

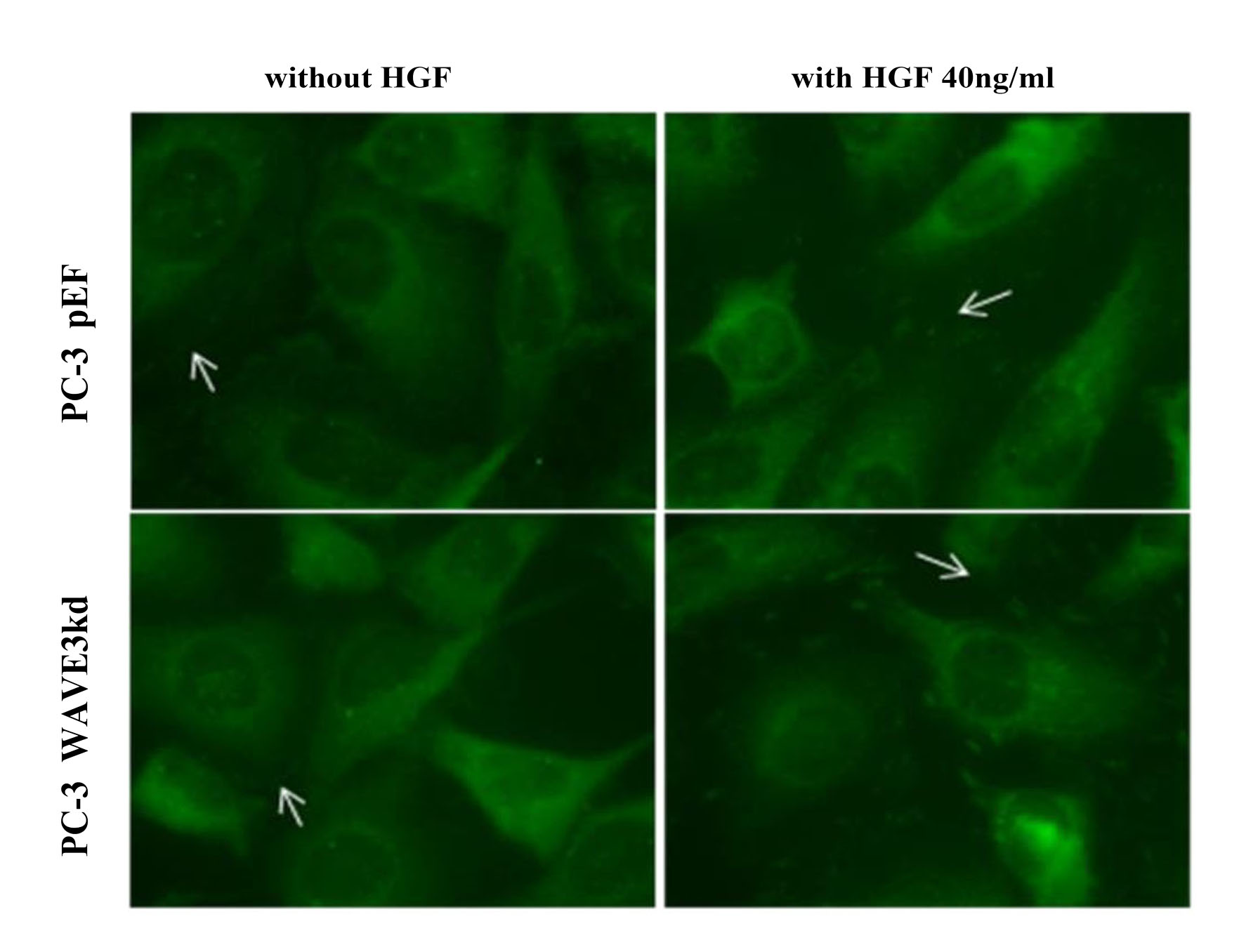

Supplement: Supplementary file 2 — IF of FAK in PC-3 cells in exposure to HGF (40 ng/ml) using an anti-FAK antibody (BD Biosciences, 610087). A FITC tagged secondary antibody was used for the IF. Arrows point to staining of FAK. [file 12935_2015_203_MOESM2_ESM.jpeg]

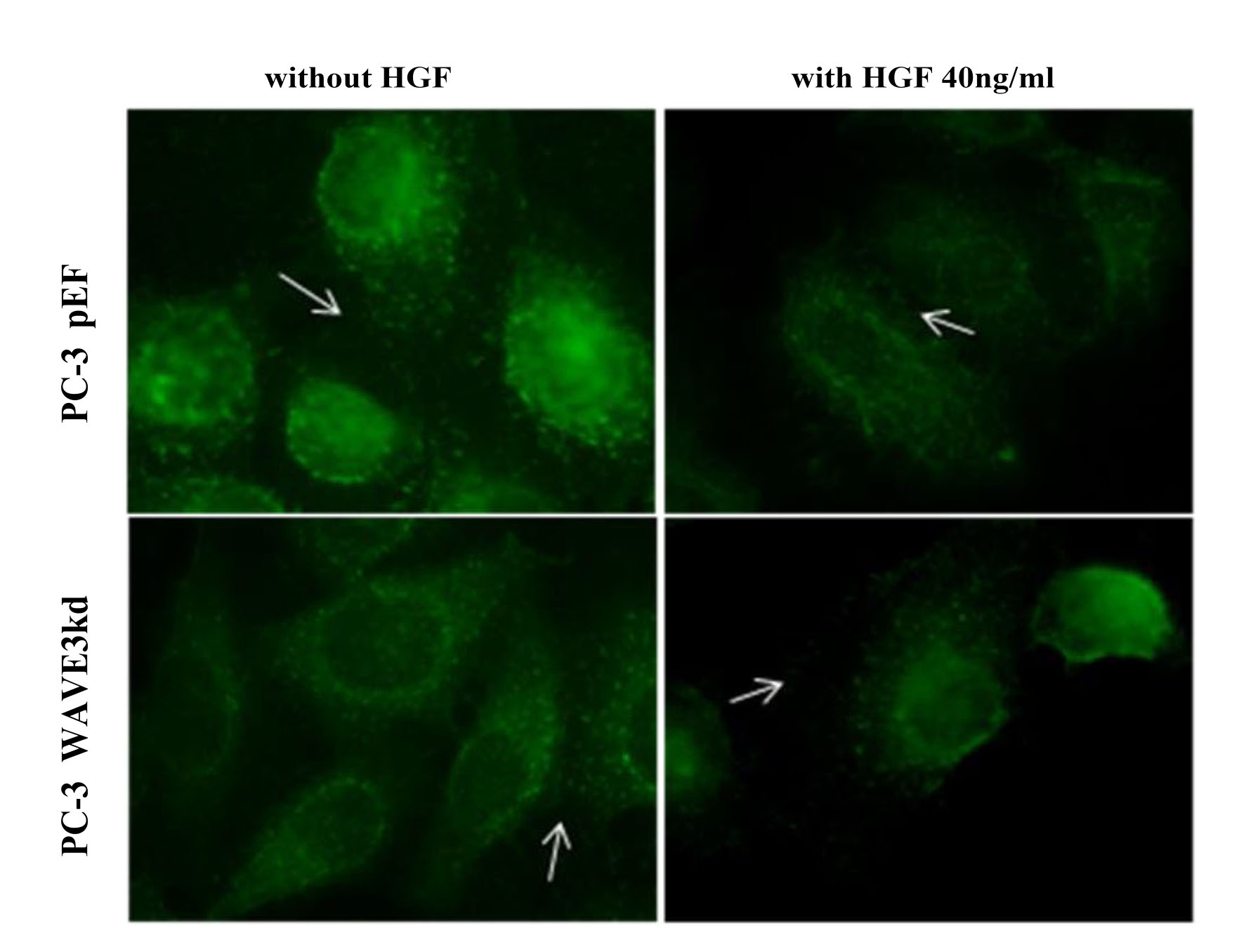

Supplement: Supplementary file 3 — Radixin in PC-3 cells in exposure to HGF (40 ng/ml) was stained using an anti-Ezrin antibody (Santa Cruz, SC-6408) and a FITC tagged secondary antibody. Arrows point to staining of Radixin. [file 12935_2015_203_MOESM3_ESM.jpeg]

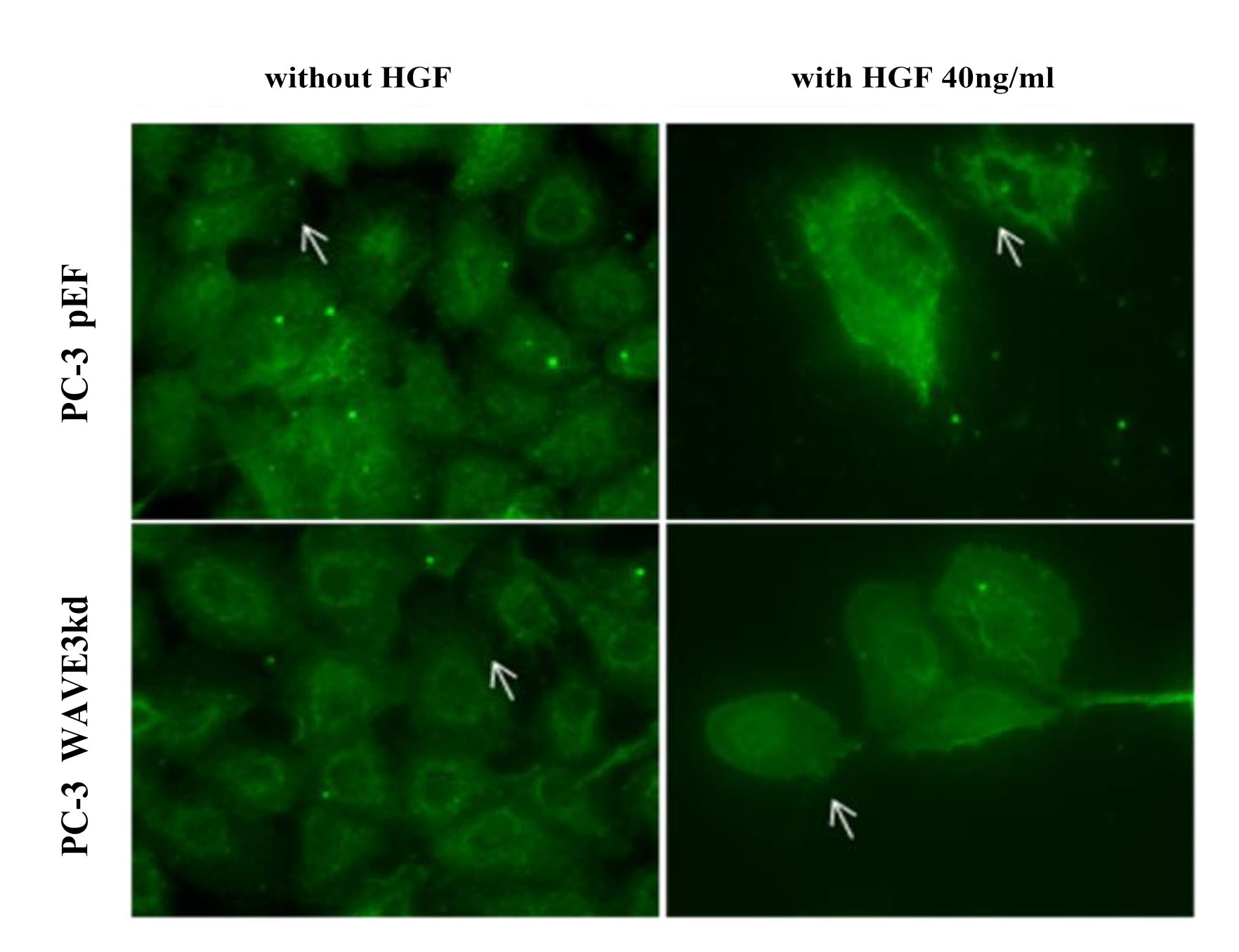

Supplement: Supplementary file 4 — IF was performed for Moesin in PC-3 cells in exposure to HGF (40 ng/ml) using an anti-Ezrin antibody (Santa Cruz, SC-13122) and a FITC tagged secondary antibody. Arrows point to staining of Moesin. [file 12935_2015_203_MOESM4_ESM.jpeg]
